# Head and neck squamous cell carcinomas of unknown primary: Can ancillary studies help identify more primary tumor sites?

**DOI:** 10.1016/j.yexmp.2024.104915

**Published:** 2024-07-03

**Authors:** Troy Hutchens, Wade Thorstad, Xiaowei Wang, Yuanxiang Li, Eric J. Duncavage, Lulu Sun, Rebecca D. Chernock

**Affiliations:** a Department of Laboratory Medicine & Pathology, University of Washington, Seattle, WA, United States of America; b Department of Radiation-Oncology, Washington University School of Medicine, St. Louis, MO, United States of America; c Department of Pharmacology and Regenerative Medicine, The University of Illinois at Chicago, Chicago, IL, United States of America; d Department of Pathology and Immunology, Washington University School of Medicine, St. Louis, MO, United States of America; e Department of Otolaryngology Head and Neck Surgery, Washington University School of Medicine, St. Louis, MO, United States of America

**Keywords:** Squamous cell carcinoma, cancer of unknown primary, Mutational signature analysis, UV mutational signature, Human papilloma virus, Epstein–Barr virus

## Abstract

A subset of head and neck squamous cell carcinomas present solely as metastatic disease in the neck and are of unknown primary origin (SCCUP). Most primary tumors will ultimately be identified, usually in the oropharynx. In a minority of cases, the primary site remains elusive. Here, we examine the role of ancillary testing, including mutational signature analysis (MSA), to help identify likely primary sites in such cases. Twenty-two cases of SCCUP in the neck, collected over a 10-year period, were classified by morphology and viral status; including human papillomavirus (HPV) testing by p16 immunohistochemistry (IHC) and RT-qPCR, as well as Epstein-Barr virus (EBV) testing by EBER-ISH. CD5 and c-KIT (CD117) IHC was done to evaluate for possible thymic origin in all virus-negative cases. Whole exome sequencing, followed by MSA, was used to identify UV signature mutations indicative of cutaneous origin. HPV was identified in 12 of 22 tumors (54.5%), favoring an oropharyngeal origin, and closely associated with nonkeratinizing tumor morphology (Fisher’s exact test; *p* = 0.0002). One tumor with indeterminant morphology had discordant HPV and p16 status (p16+/HPV−). All tumors were EBV-negative. Diffuse expression of CD5 and c-KIT was identified in 1 of 10 virus-negative SCCUPs (10%), suggesting a possible ectopic thymic origin rather than a metastasis. A UV mutational signature, indicating cutaneous origin, was identified in 1 of 10 (10%) virus-negative SCCUPs. A cutaneous auricular primary emerged 3 months after treatment in this patient. Primary tumors became clinically apparent in 2 others (1 hypopharynx, 1 hypopharynx/larynx). Thus, after follow-up, 6 tumors remained unclassifiable as to the possible site of origin (27%). Most SCCUPs of the neck in our series were HPV-associated and thus likely of oropharyngeal origin. UV signature mutation analysis and additional IHC for CD5 and c-KIT for possible thymic origin may aid in further classifying virus-negative unknown primaries. Close clinical inspection of hypopharyngeal mucosa may also be helpful, as a subset of primary tumors later emerged at this site.

## 1. Introduction

Squamous cell carcinoma (SCC) is the most common cancer of the head and neck and can arise from a wide variety of mucosal and cutaneous sites. A subset of patients present solely with metastatic disease in the neck with a difficult to locate primary tumor. Tumors are classified as true SCC of unknown primary (SCCUP) when the primary is not identified even after a broad clinical evaluation, often including physical exam, imaging, and surgical and pathologic evaluation of suspected primary sites. These advancements in diagnostic modalities has led to a significant increase in the number of successfully identified primary sites following initial presentation (Larsen et al., 2021). True SCCUPs are estimated to account for approximately 2–5% of all head and neck cancers (Geltzeiler et al., 2017; Grau et al., 2000; Lee et al., 2020; Marur and Forastiere, 2008; Ota and Kitahara, 2021; Schmalbach and Miller, 2007; Waltonen et al., 2009).

Additional steps can be taken to classify true SCCUP to the most likely site of origin even when the primary tumor cannot be found. Viral status is complementary to clinical workup in the identification of primary tumors and is critical and routinely used for classifying SCCUP (Cheol Park et al., 2017). Recent approaches to standardize the clinical workup of SCCUPs have been aimed at streamlining diagnosis for rapid initiation of appropriate treatment (Manoharan et al., 2024). Integration of any potential additional laboratory of histopathologic diagnostic modalities into current clinical workup algorithms is essential for furthering this goal. Human papillomavirus (HPV) testing is recommended for all level II/III SCCUPs. HPV-associated SCCUP is presumed to be, and treated as, an occult oropharyngeal primary. Although much less common in the United States, Epstein-Barr virus (EBV)-associated SCCUP suggests the nasopharynx or salivary gland as the most likely site of origin. EBV testing is suggested in HPV-negative SCCUP (Maghami et al., 2020).

Cutaneous metastases may be clinically favored in patients with a significant history of non-melanoma skin cancer, who are of advanced age, male sex, and lack traditional risk factors of mucosal SCC, namely tobacco and alcohol exposure. Cutaneous metastases may also be clinically favored based on lymph node site/pattern, as specific lymph node groups have been shown to be associated with cutaneous head and neck malignancies, including parotid, external jugular, suboccipital, and postauricular (Creighton et al., 2016). Recognition of cutaneous metastases is clinically significant because surgery and radiation of mucosal sites can be avoided (Maghami et al., 2020). More recently, mutational signature analysis (MSA) has proved a reliable tool to identify tumors of cutaneous origin (Lawrence et al., 2020; Sun et al., 2019; Yang et al., 2020). Mutational signatures associated with ultraviolet (UV) radiation exposure were some of the earliest and best characterized mutational signatures and are specific to cutaneous malignancies (Brash, 2015). Although rates can vary considerably by region (based on local UV indices and other etiologic exposures), UV radiation exposure has been implicated in around 90% of cutaneous SCCs and basal cell carcinomas (Kim and He, 2014; Teng et al., 2021), 60–70% of cutaneous melanomas (Armstrong and Kricker, 1993), and 20% of Merkel cell carcinomas (Wong et al., 2015). Although MSA has the potential to identify tumors of cutaneous origin in the setting of an unknown primary, its utility in head and neck SCCUP has not been examined (Chan et al., 2019).

Lastly, in the setting of SCCUP, tumors could potentially represent primary rather than metastatic disease. One theoretical explanation for a primary SCC in the neck is origination from ectopic thymic tissue. Possible thymic origin has not been examined in SCCUPs.

Here, we performed a retrospective clinical and pathologic analysis of patients with non-parotid SCCUP who were treated at a single institution where a thorough search for a primary tumor was undertaken, but a primary tumor was not identified. Parotid SCCUP was excluded because they are almost always cutaneous in origin. Ancillary testing including the evaluation of viral status (HPV and EBV), expression of CD5 and c-KIT as a possible indication of ectopic thymic origin, and MSA (particularly to assess for UV mutational signatures) was performed to determine their clinical utility in localizing a probable tumor origin. Ancillary testing was compared to the clinical assessment of likely primary sites in addition to the subsequent emergence of any primary tumors during clinical follow-up.

## 2. Materials and methods

### 2.1. Case selection and review

This study was completed following institutional review board approval from the Human Research Protection Office at Washington University in St. Louis (IRB ID #: 202006024). Informed consent was not required for this type of retrospective study. Cases were selected from an existing database of head and neck cancer patients maintained by the Department of Radiation Oncology. Thus, all patients received radiation therapy. Patients with metastatic SCC diagnosed over a 10-year period (2007–2017) and determined to be of unknown origin after broad clinical, radiologic, and pathologic evaluation failed to identify a primary site at the time of initial diagnosis were included in the study. Metastases to the parotid region, which are usually from a cutaneous site, were not included. Additionally, patients with a history cutaneous squamous cell carcinoma of the head and neck were excluded, as these cases were clinically suspected to represent metastatic disease from cutaneous sites rather than true SCCUP. Cases without an in-house biopsy or excision specimen were excluded due to a lack of material available for histologic review and ancillary testing.

Hematoxylin and eosin (H&E) stained slides were reviewed and the metastases were classified as keratinizing, nonkeratinizing, nonkeratinizing with maturation, or indeterminate, as previously described (Gondim et al., 2016). Fisher’s exact test was used to determine if there was a significant association between morphology and HPV results (assessed separately for p16 IHC and HPV RT-qPCR). Other pathologic features were obtained from pathology reports including lymph node level(s) involved, number of tumor deposits, and size of the metastasis. Clinical data including patient demographics, diagnostic evaluation, history of exposure to carcinogens, and clinical follow-up including the emergence of possible primary tumors at a later date were obtained from the electronic medical record. The mean length of follow-up from the start of treatment was 10.8 years (2.9 SD) (range 5.7–15.3 years).

### 2.2. Nucleic acid extraction and whole exome sequencing

Areas of tumor were outlined on the H&E slides for accurate macroscopic tumor identification. Tumor and paired non-tumor tissues were collected by coring the corresponding areas of formalin-fixed paraffin-embedded (FFPE) blocks using a 1 mm biopsy punch. A total of 6–7 cores were collected from each tumor and non-tumor block for nucleic acid extraction. Nucleic acid extraction (DNA and RNA), whole exome sequencing, and initial bioinformatic analysis were performed by the Genome Technology Access Center (GTAC) at the McDonnell Genome Institute (MGI). Genomic DNA and total RNA were extracted from cores of FFPE tissue using AllPrep DNA/RNA FFPE Kits (Qiagen, Germantown, MD, USA) following standard protocols.

Genomic DNA samples were quantified using a Qubit Fluorometer 3.0 or a VarioSkan Flash. 100–250 ng of gDNA was fragmented using a Covaris LE220 to achieve a mean fragment size of about 200–375 bp. Libraries were then constructed using the KAPA Hyper Prep Kit (KAPA Biosystems, Wilmington, MA, USA) on a Perkin Elmer SciClone G3 NGS automated workstation (96-well configuration). DNA libraries were pooled at an equimolar ratio and hybridized with the xGen Exome Research Panel v1.0 reagent (IDT Technologies, Coralville, IA, USA) capture reagents covering a 39 Mb target region (19,396 genes) of the human genome. The libraries contain custom Illumina adapters with 10 base dual unique indexes. The resulting library fragments were loaded onto an Illumina NovaSeq-6000 S4 flow cell and sequenced from each end (paired-end reads) for 150 bases. Libraries were demultiplexed using bcl2fastq software.

Samples were processed using a local DRAGEN server (running version 07.021.609.3.9.3 of the DRAGEN software) in somatic (tumor/normal) mode using a GRCh38 human reference. Alignments were output in CRAM format and duplicate reads were marked prior to variant calling. Short variants passing all quality thresholds and localized to the exome target region were extracted and annotated using ANNOVAR.

### 2.3. HPV RT-qPCR and EBER ISH

HPV status by quantitative reverse transcription PCR (RT-qPCR) was performed on extracted RNA and the expression of the E6 and E7 oncogenes was assessed in 13 high-risk HPV subtypes (16, 18, 31, 33, 35, 39, 45, 52, 56, 58, 59, 66, and 68) as previously described (Gao et al., 2012). EBER ISH was performed on all HPV RT-qPCR negative tumors using formalin-fixed, paraffin-embedded 4 μm tissue sections with an I View Blue Plus Detection Kit (Ventana Medical Systems, Tucson, AZ, USA). The assays used the Ventana INFORM EBER probe (Ventana Medical Systems). Staining was in an enclosed system and was performed according to the manufacturer’s instructions. Ventana Red Counterstain II (Ventana Medical Systems) was used. Positive staining was identified as blue nuclear dots.

### 2.4. Immunohistochemistry

IHC for p16 (E6H4 clone, CINtec; Ventana Medical Systems), CD5 (SP19 clone, Ventana Medical Systems), and c-KIT (YR145 clone, Cell Marque, Rocklin, CA, USA) was performed using FFPE tissue cut at 4 μm tissue sections on a Ventana BenchMark automated immunostainer by standard protocols with adequate controls. Antigen retrieval, standard on the machine, used the Ventana Ultra CC1, EDTA-Tris pH 8.0 solution (Ventana Medical Systems). p16 IHC was performed on the subset of patients, prior to 2009, for whom p16 IHC was not performed routinely for patient care. For patients diagnosed after 2009 when p16 IHC became standard of care at this institution, the existing p16 immunostain (E6H4 clone) was reviewed. College of American Pathologists (CAP) guidelines were followed for the categorization of p16 expression as positive or negative (Lewis et al., 2018). Positive staining was defined as diffuse nuclear and cytoplasmic staining in at least 70% of tumor cells and of at least moderate intensity. CD5 and c-KIT IHC was performed on the subset of virus-negative cases (as determined by HPV RT-qPCR and EBER ISH).

### 2.5. Mutational signature analysis

MSA was performed on the SCCUPs and three cases of known cutaneous SCC from sun-exposed sites as controls for UV radiation-induced squamous cell carcinoma using the web application mSigAct (Mutational Signature Activity) (Boot et al., 2018; Ng et al., 2017), a tool for plotting mutational spectra and identifying specific mutational signatures that likely contribute to these mutational spectra. Variant Call Format (VCF) files composed of high-confidence somatic mutations were used to plot single base substitution (SBS) and doublet base substitution (DBS) mutational spectra. For DBS mutational spectra composed of ≤5 DBSs, mutational signature attribution was not performed given the limited dataset. Reconstructed spectra, composed of select specific mutational signatures and their relative contributions, were created and compared to the plotted mutational spectra with similarity estimated by cosine similarity analysis. The relative contributions of specific SBS and DBS Catalogue of Somatic Mutations in Cancer (COSMIC)-reported mutational signatures (v3.2) were estimated for the plotted mutational spectra. The contribution of specific mutational signatures represents the fraction that specific mutational signature spectra contribute to the reconstructed mutational spectrum and provides an estimate of their contribution to the plotted mutational spectrum and the underlying biological mutational processes. Cases were classified based on their primary mutational signature contributions for both SBS and DBS COSMIC-reported mutational signatures, where the primary mutational signatures represents the mutational signature with the largest contribution to the reconstructed mutational spectra.

## 3. Results

### 3.1. Clinical presentation

A total of 22 cases of SCCUP were identified over the 10-year period out of 1281 patients in the head and neck cancer database (1.7%). The clinical work-up was variable for each case, but generally included a thorough medical history, an extensive head and neck examination, multiple diagnostic imaging modalities, and additional biopsies/surgical excisions with histologic examination, when appropriate. All but one patient received a PET/CT scan has part of the initial diagnostic evaluation. The remaining patient received a CT scan of the head and neck as well as a CT scan of the chest. All patients with level II/III lymph node metastases underwent surgical resection of oropharyngeal tonsillar tissue in a search for the primary tumor, except for 3 patients. One of the 3 patients was considered to have poor endoscopic access and 2 were treated early in the study period (2007), likely prior to the establishment of surgical resection as standard of care. The clinical features of the patients are detailed in Table 1. The mean age at the time of treatment was 58.4 years (8.6 SD) with an age range of 43–77 years. The majority of SCCUP cases were identified in male patients (77.3%) as compared to female patients (22.7%).

SCCUP presented as a solitary metastasis in 68.2% and as multiple metastases in 31.8% of patients. Level II was the most commonly involved lymph node group (18 of 22 or 81.8%). The number of separate tumor deposits in cases with multiple metastases ranged from 2 to 9 separate deposits. The mean size of the largest tumor deposit(s) was 4.3 cm (1.9 SD) with a range of 1.7–11 cm. Only 1 patient (4.5%) presented with bilateral neck disease.

### 3.2. Histopathologic features and viral status

**Fig. 1. –:**
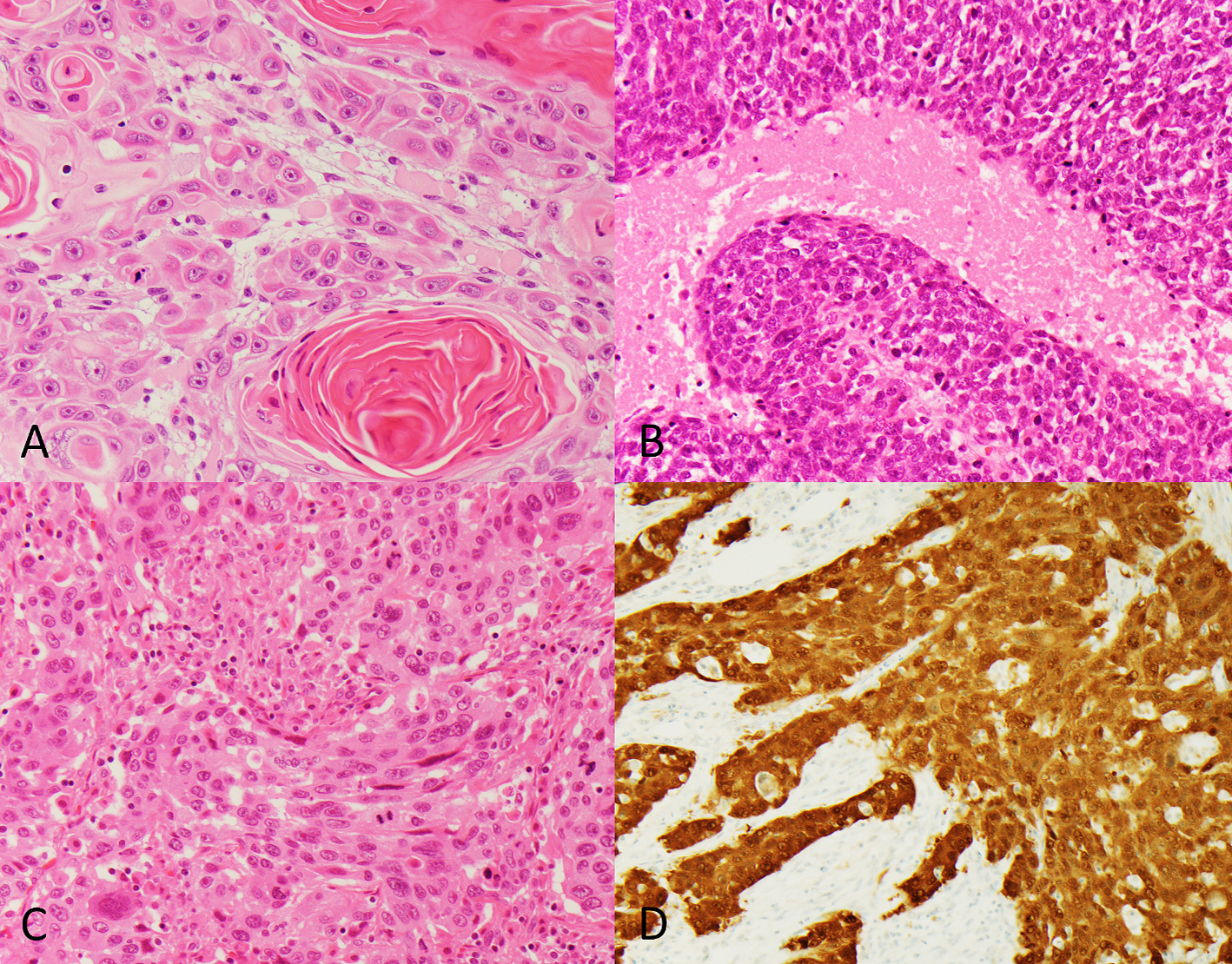
Representative tumors showing keratinizing **(A)**, nonkeratinizing **(B)**, and indeterminate **(C)** morphologies (images at 400× magnification). Positive p16 immunostaining in tumor with indeterminate morphology (HPV-negative) (200× magnification) **(D)**.

Histologic features and HPV testing results for the case series are summarized in Table 2. Twice as many SCCUP cases showed nonkeratinizing morphology (including cases with maturation) as showed keratinizing morphology (63.6% versus 31.8%). Only a single case displayed indeterminate morphology, best characterized as keratinizing versus nonkeratinizing with extensive maturation. Representative images of the tumor morphologies are shown in Fig. 1.

HPV RNA was identified by RT-qPCR (a ‘gold standard’ method for HPV detection) in 12 of 22 (54.5%) of SCCUPs. HPV16 was detected in all 12 HPV-positive SCCUPs with a single tumor showing co-expression of both HPV16 and HPV33. All HPV-positive cases were additionally positive for p16 IHC. One case with indeterminate morphology was p16-positive but HPV-negative. Thus, p16 IHC showed a sensitivity of 100% and a specificity of 90.0%, as compared to HPV RT-qPCR.

There was a statistically significant association between tumor morphology and HPV results as separately assessed by p16 IHC (*p* = 0.00031, two-sided) and HPV RT-qPCR (*p* = 0.00024, two-sided) (Table 2). A single case of nonkeratinizing SCCUP (10%) and a single case of nonkeratinizing SCCUP with maturation (25%) were negative for p16 and HPV. All keratinizing SCCUPs were p16 and HPV-negative. EBV by EBER-ISH was not detected in any HPV-negative tumor.

### 3.3. Thymic marker expression

Concurrent diffuse expression of CD5 and c-KIT was identified 1 of 10 (10%) of virus-negative SCCUPs (case 13). This patient presented with what was clinically/radiologically through to be a left level IIA lymph node and showed keratinizing-type squamous cell carcinoma with monotonous cytology, basaloid features, and a dense lymphocytic infiltrate (Fig. 3). A capsule was present at the periphery suggestive of possible residual lymph node. The remaining tumors were both CD5 and c-KIT negative, including one case that showed clinical and pathologic features suspicious for thymic origin (soft tissue metastasis near the thyroid with broad fibrous bands, monotonous tumor cell population, and lymphocytic infiltration). Histology reminiscent of thymic carcinoma was not present in any other SCCUP.

### 3.4. Whole exome sequencing and mutational signature attribution

Whole exome sequencing on tumor samples showed an average depth of coverage of 188× (range: 41–308, median: 187). Whole exome sequencing on paired normal samples showed an average depth of coverage of 87× (range: 23–256, median: 65). SCCUP cases showed a mean of 644 separate SBSs (total count per tumor) (428 SD) with a range of 127–1891; while the cutaneous SCC controls showed a higher mean of 6218 SBSs (total count per tumor) (1985 SD) with a range of 3526–8253, as expected for cutaneous carcinomas, which are known to have a high mutational burden. The preponderate SBS mutational signature attributed to SCCUP cases was SBS1, corresponding to spontaneous deamination of 5-methylcytosine (clock-like signature). This SBS1 mutational signature was the primary attributed SBS mutational signature in 19 of 22 cases (86.4%), regardless of HPV status and included the SCCUP that showed diffuse CD5 and c-KIT expression, suggestive of possible thymic origin.

Three SCCUP cases (14, 15, and 17) showed an alternative primary attributed SBS mutational signature. For case 14, the primary attributed SBS mutational signature was SBS7b (ultraviolet light exposure). For case 15, the primary attributed SBS mutational signature was SBS6 (defective DNA mismatch repair) and for case 17, the primary attributed SBS mutational signature was SBS87 (thiopurine chemotherapy treatment). SBS7b (ultraviolet light exposure) was the primary attributed SBS mutational signature for all of the cutaneous SCC controls.

A significant number of separate DBSs (76 in total) were identified in only a single case (case 14). In the remaining SCCUP cases, 5 or fewer DBSs were identified per case (including many cases without any DBSs) and therefore mutational signature attribution was not performed. All of the DBSs in case 14 were attributable to ‘CC to TT’ mutations and the primary attributed DBS mutational signature was DBS1 (ultraviolet light exposure). The cutaneous SCC controls showed a mean of 201 DBSs (total count per tumor) (52 SD) with a range of 138–265. DBS1 (ultraviolet light exposure) was the primary attributed DBS mutational signature for all of the cutaneous SCC controls.

The presence of UV signature mutations in case 14 was corroborated by the clinical history. At the time of SCCUP diagnosis, a cutaneous primary was suspected but was not identified. A cutaneous primary site was favored due to the patient’s significant clinical history of UV light exposure and personal history of multiple skin cancers; including basal cell carcinoma of the arm, face, and back, and squamous cell carcinoma of the leg and hand. There was no history of SCC of the head and neck at the time of diagnosis. Three months following the initial diagnosis of metastatic cancer, a primary cutaneous SCC emerged on the patient’s posterior ear (ipsilateral to the SCCUP).

Additional support for the primary attributed SBS mutational signatures identified in case 15 (SBS6, defective DNA mismatch repair) and case 17 (SBS87, thiopurine chemotherapy treatment) were not identified upon further review of the medical record. A limited number of mutations were identified in case 17, resulting in a weak mutational spectrum and non-robust MSA. The secondary mutational signature identified in case 15 was SBS1 (spontaneous deamination of 5-methylcytosine) with a contribution of 0.29 (data not shown) was the primary attributed SBS mutational signature in the majority of SCCUP cases. Mutational signature attribution in these cases is thought to be of minimal interpretive value due to inadequate mutational data and a lack of supporting clinical information. The results of the MSA are summarized in Supplemental Table 1.

### 3.5. Emergence of primary tumors

**Fig. 2. –:**
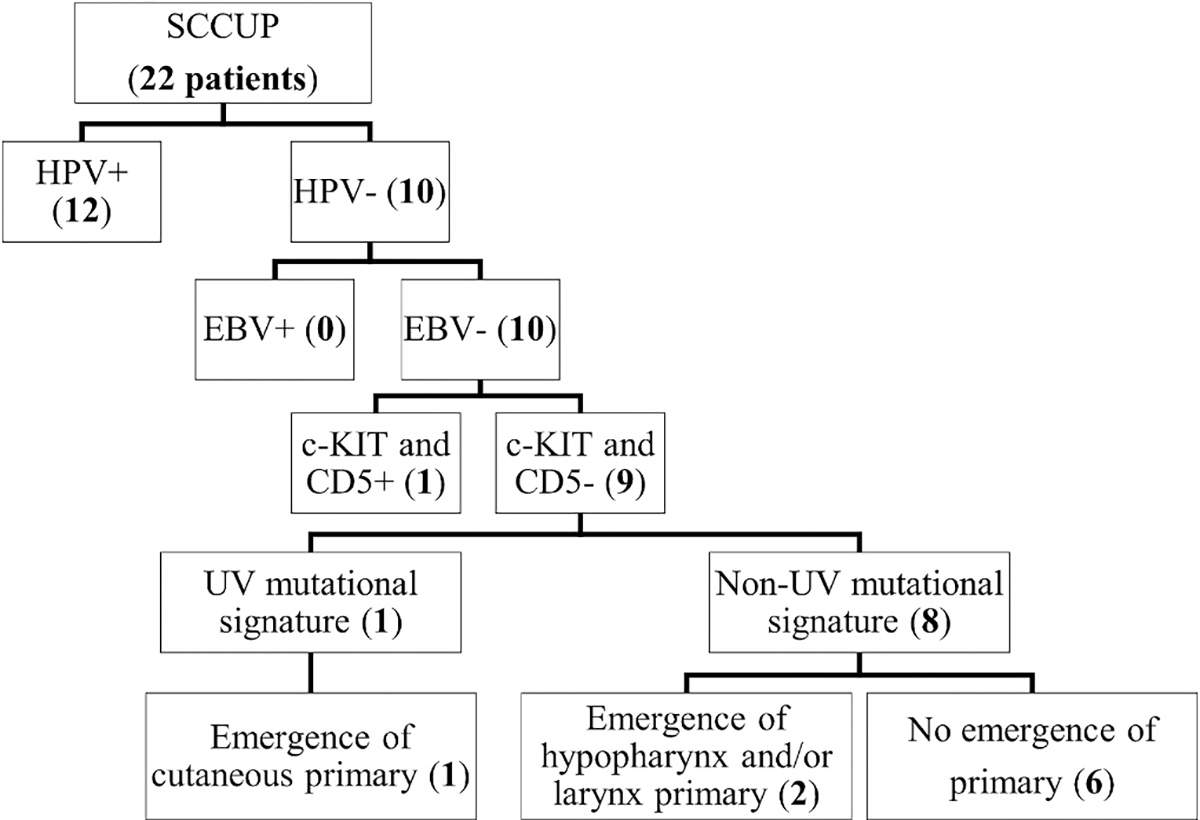
Categorization and breakdown of all SCCUP cases by viral testing (HPV and EBV), IHC (c-KIT and CD5), MSA, and eventual emergence of a primary site.

**Fig. 3. –:**
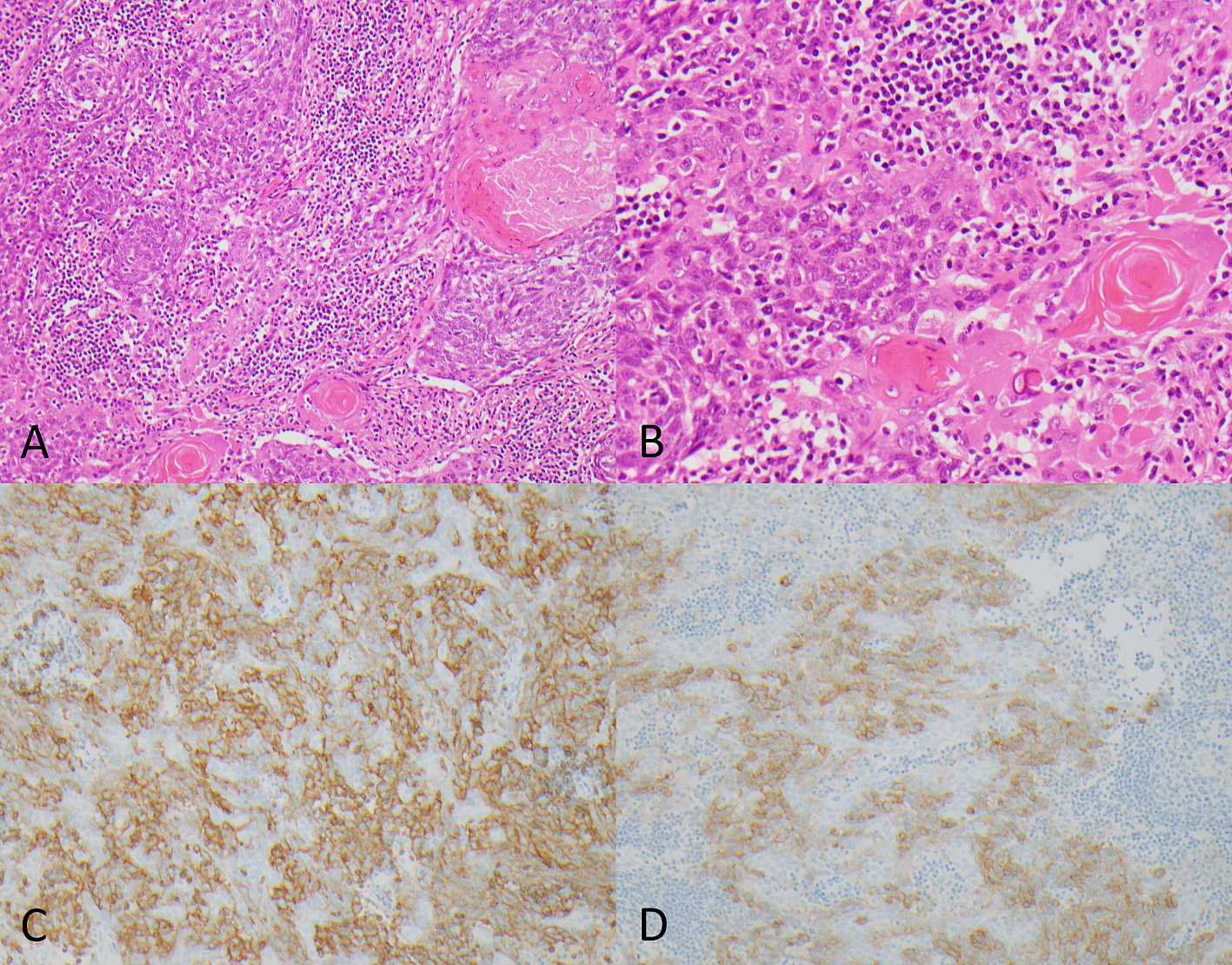
Representative images of case 13 showing keratinizing-type squamous cell carcinoma at 100× magnification **(A)** and 200× magnification **(B)** with relatively monotonous cytological features and a dense lymphocytic infiltrate (panel B represents a magnified portion of panel A). This case showed diffuse positive staining for both CD5 **(C)** and c-KIT **(D)**, raising the possibility of thymic origin (images at 200× magnification).

Besides the patient with the emergence of a cutaneous (posterior ear) SCC described above, potential primary head and neck SCCs were subsequently identified in two additional patients (1 in the hypopharynx and 1 in the larynx/hypopharynx), both with virus-negative SCCUP. Case 19 initially presented with bilateral neck masses and 17 months later the patient was found to have a SCC of the posterior hypopharynx involving the midline. Bilateral cervical metastases are often associated with a primary tumor involving midline structures and unilateral cervical metastases are often associated with a primary tumor involving the ipsilateral side. However, this is not always true. Case 16 initially presented as a single right cervical metastasis and 8 months later an SCC of the contralateral left pyriform sinus/supraglottis was identified. At the time of surgical resection of the primary hypopharyngeal/laryngeal tumor, a left neck dissection revealed metastatic SCC present in the ipsilateral left level II lymph nodes. Generally, laterality is utilized to narrow the field of additional surgical interventions (excisions and biopsies) performed to potentially identify an occult primary. In case 16, the patient underwent ipsilateral tonsil/base of tongue resection and biopsies of the ipsilateral glossotonsillar sulcus, pyriform sinus, and vocal cord but ultimately a primary emerged on the contralateral side. Of note, a third patient developed a left oral tongue SCC 7 years after originally being diagnosed with a left neck SCCUP. Due to the extended length of time between the two diagnoses, two separate and unrelated processes were clinically favored. The distribution of cases by viral status, IHC, MSA, and the emergence of likely primary sites is summarized in Fig. 2.

At last follow-up, 5 patients were deceased (22.7%) with at least 2 deaths due to disease progression (9%). Of the remaining patients, 16 were still alive (72.7%) and 1 was without available follow-up (4.5%). One of the deaths occurred in a patient with eventual primary emergence in the hypopharynx. Of the 5 patient deaths identified, 4 of them were in HPV-negative SCCUP (including 2 deaths due to disease progression and 2 deaths without disease) and 1 was associated with an HPV-positive SCCUP (death without disease).

## 4. Discussion

Current recommendations for the evaluation of a patient with head and neck SCCUP include extensive clinical/surgical, radiologic, and pathologic studies to identify a primary tumor or narrow down likely primary sites. These include, but are not limited to, a thorough medical history and physical examination including flexible endoscopy, contrast-enhanced computed tomography and potentially positron emission tomography, assessment for HPV in level II and III metastases and potentially EBV, and additional surgical biopsies and resections when clinically appropriate (Maghami et al., 2020; Podeur et al., 2020). This intensive type of investigation has been shown to result in the identification of a primary tumor site in anywhere from 50 to 90% of cases (Lee et al., 2020; Lewis et al., 2018). The rates of primary site identification are dependent on variations in clinical workup, including the extent of surgical procedures and sites examined.

Clinical presentation, distribution/pattern of lymph node metastases, and viral status can also provide clues to the likely location of a primary tumor. Specific primary sites show different metastatic frequencies and affect different lymph node sites and levels (Kountakis, 2013; Shah, 1990). Level II/III metastases most commonly originate from the oropharynx and are frequently HPV-associated. HPV status in level II/III metastases is important not only to support likely oropharyngeal origin but also because HPV status is a prognostic indicator and affects the treatment and management of SCCUP. With current treatment approaches, HPV-associated SCCUP has a similar favorable prognosis to primary HPV-associated oropharyngeal SCC (Cheraghlou et al., 2019; Ota and Kitahara, 2021; Ross et al., 2018). No patient with HPV-associated SCCUP died of disease in our series. The best method to determine HPV status in SCCUP remains controversial. The CAP currently recommends p16 IHC as a surrogate marker for HPV when the metastasis is located in a level II/III lymph node and the tumor morphology is nonkeratinizing. In contrast, the American Society of Clinical Oncology (ASCO) recommends HPV-specific testing in all p16-positive tumors (Maghami et al., 2020). Here, we found that 1 of 22 (4.5%) SCCUPs had discordant p16 and HPV tumor status. The discordant tumor had indeterminate morphology and therefore would have undergone HPV testing by either the CAP or ASCO recommendations. Nevertheless, HPV-specific testing for confirmation of positive p16 IHC may be warranted in the setting of an unknown primary metastasis as p16 loses specificity for HPV at non-oropharyngeal sites (Lewis et al., 2018). Strong diffuse nuclear and cytoplasmic expression of p16 can be seen in up to 32% of squamous cell carcinomas of cutaneous origin, independent of high-risk HPV (Satgunaseelan et al., 2017). Additionally, strong p16 expression has been reported in 8–14% primary lung squamous cell carcinomas, independent of high-risk HPV (Schulte et al., 2020; Zhou et al., 2019).

Over half of the SCCUP cases in this study were HPV-associated and therefore likely of oropharyngeal origin. None were EBV-positive in this North American patient population. The rate of HPV-associated head and neck SCCUP reported here (54.5%) is relatively consistent with, although slightly higher than, previously reported rates in similar case series (37–41%) (Lee et al., 2020; Tribius et al., 2012). Interestingly, one patient in our series had a co-infection of HPV16 and HPV33, which is rare in head and neck cancers. No other cases of multi-infection have been identified by the described RT-qPCR methodology in the close to 900 previously tested cases of oropharyngeal squamous cell carcinoma (Gao et al., 2012; Lewis et al., 2021; Shinn et al., 2021). It is important to note that RT-qPCR, in addition to being a sensitive methodology, is also highly specific (Gao et al., 2012) and comparable to HPV RNA ISH, which is more commonly used in clinical practice(Gao et al., 2012) and comparable to HPV RNA ISH, which is more commonly used in clinical practice. Other methods of HPV detection, particularly some DNA PCR tests, are sensitive, but less specific and are prone to high rates of false positives due to environmental/laboratory contamination. Thus, previously reported cases of co-infection in head and neck HPV-associated SCC may be attributable to method choice rather than in-vivo biology.

While unusual in head and neck squamous cell carcinoma, multi-infection with more than one HPV type is relatively common in SCC of the uterine cervix. Multi-infection rates in cervical disease are estimated to be between 26% and 43% of all HPV-positive cases (Chaturvedi et al., 2011; Liao et al., 2020). In certain populations, the rate of multi-infection is higher than the rate of mono-infection, at a rate of 75% of HPV-positive cases (Gallegos-Bolaños et al., 2017). Additionally, multi-infection in cervical disease has been linked to persistent HPV infection of longer duration and a stronger association with HSIL (Kim et al., 2021). It is unclear if any of these findings translate to head and neck HPV-associated SCC given the rarity of multi-infection in head and neck sites.

HPV status was additionally confirmed utilizing the available whole exome sequencing data. All HPV-positive SCCUPs demonstrated multiple 50 base pair alignments with the evaluated HPV genomes present in the GRCh83 reference. Interestingly, a single HPV-negative SCCUP (case 21) demonstrated low alignment counts with HPV26. The significance of this finding is uncertain, as this case was p16 positive by immunohistochemistry, but negative for HPV by RT-qPCR, a methodology generally considered to be highly sensitive and specific for the detection of HPV.

In virus-negative cases, we attempted to further classify SCCUP both by immunophenotypic assessment for possible thymic origin and by UV signature mutation analysis as an indicator of cutaneous origin. Neither possible thymic origin nor mutational signatures have previously been investigated in head and neck SCCUP. Mutational signatures, specifically UV mutational signatures, have, however, been used to identify other tumors that were previously of unclear origin as being of cutaneous origin; including “lung-only” melanoma (Yang et al., 2020), lymph node Merkel cell carcinoma (Lawrence et al., 2020), and salivary high-grade neuroendocrine carcinomas (Sun et al., 2019). Although whole exome sequencing data was generated for this study, it is important to note that MSA, particularly for UV mutational signatures, can be performed on smaller more accessible massive parallel sequencing panels (Pham et al., 2020). Few commercially available massive parallel sequencing panels currently report the presence of a UV mutational signature. However, the data generated from these panels is sufficient to assess for the presence of a UV mutational signature and can often be reported upon request.

Expression of CD5 and c-KIT was observed in a single case (1 of 10 HPV-negative SCCUPs) raising the possibility of thymic origin, but this is not definitive, particularly because expression of thymic markers in head and neck cancer has not been thoroughly investigated. However, CD5 expression has not been observed in lung squamous cell carcinomas among thousands of non-small cell carcinomas that have been tested (Asirvatham et al., 2014; Jha et al., 2017; Kriegsmann et al., 2015). This particular CD5/c-KIT positive tumor in the neck shows some histologic features reminiscent of thymic carcinoma but was surrounded by a fibrous capsule, suggestive of a nodal metastasis. A second non-nodal putative soft tissue metastasis located adjacent to the thyroid gland, where thymic carcinomas are known to arise, was histologically suspicious for thymic carcinoma but was negative for thymic markers by IHC. No other SCCUPs showed histologic features reminiscent of thymic carcinoma. Nevertheless, the possibility of a primary tumor, such as an ectopic thymic carcinoma, is an important consideration in the setting of an SCCUP. Ectopic thymic carcinomas are rare, but have been reported the cervical neck (largely in and around the thyroid), as well as other sites including parietal pleura, parotid, and intrapericardial(Calderon et al., 2008; Cheng et al., 2022; Wang et al., 2023; Yao et al., 2010; Zhu et al., 2017). Within the context of this case series, which excluded parotid metastases, SCCUP arising from occult cutaneous primary SCC appears to be relatively uncommon and was accompanied by additional clinical information suggestive of a cutaneous origin. Here, we found a single case of SCCUP (1 of 22 overall or 1 of 10 HPV-negative SCCUP) to harbor a UV mutational signature supporting a cutaneous origin of the metastasis. This particular tumor was clinically suspected to be of cutaneous origin given the patient’s history of skin cancers, although none were SCCs of the head and neck. Additionally, a primary cutaneous SCC of the posterior ear did subsequently emerge 3 months after diagnosis. Our mutational signature findings are concordant with both the initial clinical impression and the later identification of a cutaneous SCC. Prospective confirmation of cutaneous origin in this patient’s tumor may have spared this patient from oropharyngeal biopsy and may have led to earlier detection of the primary tumor. In other patients, identification of UV mutational signatures may help avoid overtreatment with surgery and radiation to mucosal sites. Currently, if a head and neck SCCUP presents with a clinical scenario that is highly suggestive of an occult cutaneous primary SCC, the American Society of Clinical Oncology recommends avoiding mucosal surgical approaches and radiation to mucosal sites as well as the contralateral neck (Maghami et al., 2020).

The remaining cases in our series did not harbor other mutational signatures that could help identify likely primary sites. HPV-negative SCC has been reported to be frequently characterized by C > A mutations associated with the SBS4 mutational signature (tobacco smoking). In this case series, contributions from the SBS4 mutational signature were not identified in any case, regardless of HPV status (data not shown). Contributions from other mutational signatures attributable to tobacco use (SBS29 and SBS92) were similarly not present or only present as a small, likely insignificant, contribution.

HPV-positive SCCs reportedly demonstrate C > T (SBS2) and/or C > G (SBS13) mutations at TpCpN trinucleotide sites (activity of APOBEC family of cytidine deaminases) (Cannataro et al., 2019). The activation-induced deaminase/apolipoprotein B mRNA editing catalytic polypeptide-like (AID/APOBEC) family of cytidine deaminases are intrinsically able to bind to both RNA and single-stranded DNA. The AID/APOBEC family shows a wide variety of protein and tissue-specific activity including a role in innate immunity through anti-viral activity mediated by the deamination of C to U in viral RNA or single-stranded viral DNA (Riva et al., 2021). HPV infection and the expression of E6 and E7 have been shown to upregulate AID/APOBEC family proteins (Vieira et al., 2014). It is hypothesized that the overexpression of AID/APOBEC proteins contribute to the accumulation of somatic mutations and malignant progression in HPV-positive tumors through the deamination of non-viral single-stranded DNA present during cell replication (Kono and Laimins, 2021).

Despite over half of the SCCUP cases testing positive for HPV, SBS2 and SBS13 mutational signatures (activity of APOBEC family of cytidine deaminases), were not identified as a primary mutational signature in this case series. HPV-positive SCCUP cases demonstrated an increased, but not statistically significantly difference, in mean APOBEC contribution (accounting for either SBS2 and/or SBS13) as compared to HPV-negative SCCUP cases; 0.077 (0.106 SD) as compared to 0.036 (0.052 SD), respectfully. Three cases demonstrated contribution from SBS2 or SBS13 within the top 3 identified mutational signatures (data not shown). All three of these cases (2, 5, and 9) were positive for HPV. Together, these findings potentially support an association between HPV positivity and mutational changes due to the activity of the APOBEC family of cytidine deaminases, in this limited case series.

SBS1, corresponding to spontaneous deamination of 5-methylcytosine, was the primary attributed SBS mutational signature identified in the majority of SCCUP cases, regardless of tumor HPV status. SBS1 mutational signatures are generally considered ubiquitous among cancer types, including head and neck cancers (Alexandrov et al., 2015). This mutational process is endogenous and mediated by spontaneous or enzymatic deamination of 5-methylcytosine to thymine. This process is considered ‘clock-like’ in that it serves as a biological clock with the number of these mutations in a cell and the contribution of the SBS1 mutational signature in a tumor being proportional to the chronological age of the cell and patient (Alexandrov et al., 2015).

One limitation of this study is that cases were selected from a population of radiation oncology patients receiving radiotherapy. Any patients managed without the use of radiotherapy would not have been identified for inclusion in this case series, however, such cases were likely rare. Current guidelines suggest that radiotherapy can be avoided in lieu of observation only in small-volume neck disease with a single positive lymph node without histopathologic evidence of extranodal extension (Maghami et al., 2020). Thus, the observations made in this case series study may not be wholly representative of all SCCUP cases, due to potential selection bias for bilateral and/or large volume neck disease.

## 5. Conclusions

**Fig. 4. –:**
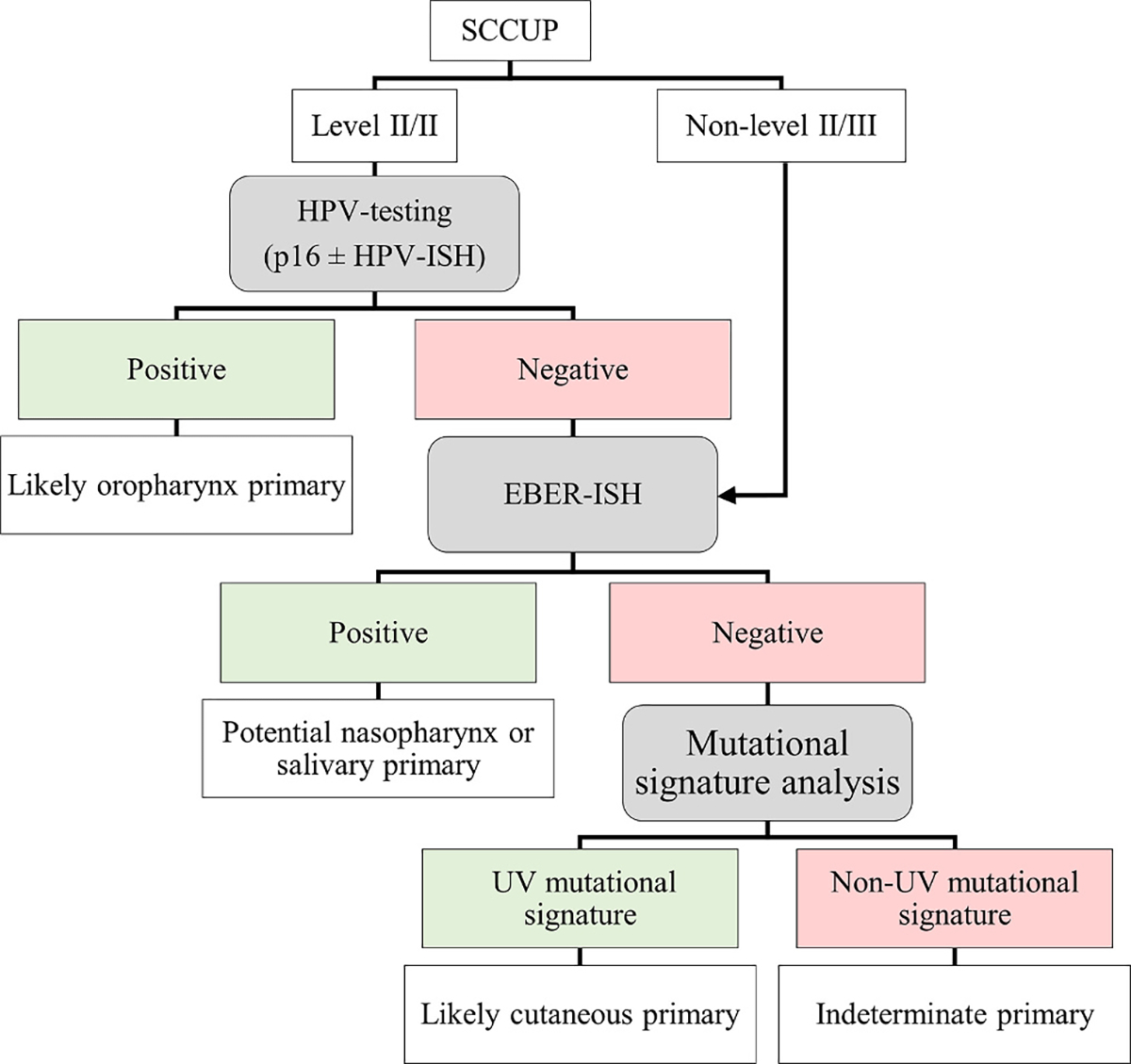
Proposed algorithm for the potential use of viral testing and MSA in the evaluation of SCCUP. MSA would likely provide the most potential benefit for viral negative level II/III lymph nodes. MSA would additionally potentially benefit cases in which there is clinical suspicion of a cutaneous primary.

In summary, 54.5% percent of SCCUP in this series were HPV-positive (favoring oropharyngeal origin), no cases were EBV-positive (favoring potential nasopharynx or salivary origin), 4.5% (a single case) was diffusely positive for c-KIT and CD5 (raising the possibility of thymic origin), and 4.5% (a single case) was identified as cutaneous based on MSA. Eight cases (36.4% of SCCUP or 0.6% of all head and neck SCCs) had no potential primary site of origin identified, however, a hypopharynx/larynx primary and a hypopharynx primary later emerged, each in one patient, ultimately leaving 6 of 22 (27.3%) with no possible site of origin identified. The above findings indicate that careful inspection of the hypopharyngeal mucosa during endoscopy is warranted even though it may be challenging to examine. In addition, MSA is available for use in routine clinical practice and may add value to the evaluation of patients with virus-negative SCCUP, as the management of cutaneous SCC is quite different from mucosal SCC. Lastly, consideration of a thymic carcinoma is suggested, especially when the tumor is located near the thyroid gland, where thymic carcinomas are known to arise. An algorithm is proposed where MSA is performed on virus-negative SCCUP cases (Fig. 4). The utility of thymic markers in SCCUP is intriguing but requires further investigation.

## Supplementary Material

1

## Figures and Tables

**Table 1:** -Clinical summary of SSCUP cases, grouped by HPV positive and HPV negative cases.

Case	Sex	Age	Tumor deposits	Laterality	Lymph node location/ level	Largest tumor deposit	Surgical workup for primary	Emergence of potential primary (location)	Length of follow-up (years)	Overall Survival (disease status)

**HPV Positive SCCUP**										
1	male	53	1	right	IIA/m	4.0	ipsilateral tonsil and base of tongue resection	No	5.7	Unknown
2	male	50	1	right	IIA/III/IV	2.0	ipsilateral tonsil and base of tongue resection	No	7.1	Alive
3	male	49	1	left	IB/IIA/III/IV	11.0	ipsilateral tonsil and bilateral base of tongue resection	No	7.3	Alive
4	female	62	1	right	II	4.0	ipsilateral tonsil and base of tongue resection	No	8.7	Alive
5	female	68	2	left	IIB and thyrohyoid	4.0	bilateral tonsil resection; ipsilateral base of tongue, nasopharynx, and soft palate biopsies	No	8.9	Alive
6	male	43	1	right	II	3.5	bilateral tonsil and ipsilateral pharynx resection; ipsilateral base of tongue biopsy	No	10.3	Alive
7	male	50	1	right	IIA	4.5	bilateral tonsil resection and ipsilateral base of tongue resection	No	10.8	Alive
8	female	74	1	right	IIB	1.7	ipsilateral tonsil and vallecula resection	No	12.0	Alive
9	male	51	1	left	I/II/III	3.5	bilateral tonsil resection; ipsilateral oral cavity sulcus, buccal mucosa, retromolar trigone, base of tongue, and tooth socket biopsies	No	12.3	Deceased (DWOD)
10	male	55	5	left	II/III	3.7	vallecula biopsy	No	12.4	Alive
11	male	55	2	right	II/III and supraclavicular	3.9	bilateral tonsil resection	No	14.1	Alive
12	male	56	4	left	III/IV/V	6.3	ipsilateral tonsil and base of tongue resection	No	8.5	Alive
**HPV Negative SCCUP**										
13	male	55	1	left	IIA	5.5	ipsilateral tonsil and bilateral base of tongue resection	No	6.5	Alive
14	male	77	1	left	V/supraclavicular	5.0	ipsilateral base of tongue biopsy	Yes (skin, left posterior ear)	7.3	Alive
15	female	74	1	right	II/III/IV	4.0	ipsilateral tonsil and base of tongue resection; ipsilateral nasopharynx biopsy	No	9.6	Deceased (DWOD)
16	female	59	1	right	II	3.0	ipsilateral tonsil resection; ipsilateral base of tongue, glossotonsillar sulcus, pyriform sinus, and vocal cord biopsies; unspecified nasopharynx biopsy	Yes (larynx, left pyriform sinus/ supraglottis)	10.2	Alive
17	male	56	4	left	IV*	3.3	hypopharynx biopsy	No	13.3	Deceased (DOD)
18	male	62	5	bilateral	parapharyngeal/II/ III/IV (right) and II/ III/IV/V (left)	5.5	left oropharynx and unspecified base of tongue resection; bilateral base of tongue, nasopharynx, and nasal cavity biopsies	No	13.4	Alive
19	male	50	1	right	II	2.3	bilateral tonsil and right base of tongue resection; right retromolar trigone and epiglottis biopsies	Yes (hypopharynx, posterior wall)	14.0	Deceased (DWOD)
20	male	59	1	right	II/III	3.5	bilateral tonsil resection; bilateral base of tongue, pyriform sinus, and nasopharynx biopsies	No	14.5	Deceased (DOD)
21	male	55	9	right	II/III/V	3.5	ipsilateral tonsil, glossotonsillar sulcus, nasopharynx, and posterior oropharynx biopsies	No	14.9	Alive
22	male	59	1	left	V	5.8	ipsilateral tonsil, base of tongue, and nasopharynx biopsies	No**	15.3	Alive

* Additional unspecified levels positive for metastatic squamous cell carcinoma.

** Patient developed left oral tongue squamous cell carcinoma 7 years after diagnosed SCCUP, favored to represent unrelated primary. Abbreviations: DWOD, died without disease; DOD, died of disease.

**Table 2:** HPV testing results of SSCUP cases grouped by morphology. Fisher’s exact tests were used to determine if there was a significant association between morphology and HPV testing results (p16 and qRT-PCR).

Morphology (n)	p16 + (IHC)	p16 − (IHC)	HPV + (qRT-PCR)	HPV− (qRT-PCR)

Nonkeratinizing (10)	9 (90%)	1 (10%)	9 (90%)	1 (10%)
Nonkeratinizing with maturation (4)	3 (75%)	1 (25%)	3 (75%)	1 (25%)
Keratinizing (7)	0 (0%)	7 (100%)	0 (0%)	7 (100%)
Indeterminate (1)	1 (100%)	0 (0%)	0 (0%)	1 (100%)
	Fisher’s Exact Test, p = 0.00031 (two-sided)		Fisher’s Exact Test, p = 0.00024 (two-sided)	

Key:

* = wildcard placeholder for all alphanumeric values, inclusive, used in ICD-10 code specifiers; all ICD-10 codes presume inclusion of the base code prior to the semi-colon (without specifiers)

## Data Availability

Data will be made available on request.
