# Supplementary material for: Head and neck squamous cell carcinomas of unknown primary: Can ancillary studies help identify more primary tumor sites?
Source: Exp Mol Pathol. Author manuscript; Available in PMC 2024 Oct 7. (PMC11458069; doi:10.1016/j.yexmp.2024.104915)
Supplement: 1 [file NIHMS2011559-supplement-1.docx]

| **Case** | **Tumor** | **Primary SBS mutational signature** | **Contribution (n)** | **Total SBS count** | **Primary DBS mutational signature** | **Contribution (n)** | **Total DBS count** |
| --- | --- | --- | --- | --- | --- | --- | --- |
| 1 | SSCUP | SBS1 | 0.51 (100) | 197 | - | - | ≤ 5 |
| 2 | SSCUP | SBS1 | 0.30 (38) | 127 | - | - | ≤ 5 |
| 3 | SSCUP | SBS1 | 0.41 (177) | 435 | - | - | ≤ 5 |
| 4 | SSCUP | SBS1 | 0.63 (546) | 873 | - | - | ≤ 5 |
| 5 | SSCUP | SBS1 | 0.25 (96) | 385 | - | - | ≤ 5 |
| 6 | SSCUP | SBS1 | 0.63 (451) | 718 | - | - | ≤ 5 |
| 7 | SSCUP | SBS1 | 0.54 (286) | 525 | - | - | ≤ 5 |
| 8 | SSCUP | SBS1 | 0.69 (931) | 1347 | - | - | ≤ 5 |
| 9 | SSCUP | SBS1 | 0.28 (154) | 545 | - | - | ≤ 5 |
| 10 | SSCUP | SBS1 | 0.44 (80) | 181 | - | - | ≤ 5 |
| 11 | SSCUP | SBS1 | 0.73 (386) | 529 | - | - | ≤ 5 |
| 12 | SSCUP | SBS1 | 0.48 (403) | 833 | - | - | ≤ 5 |
| 13 | SSCUP | SBS1 | 0.50 (201) | 401 | - | - | ≤ 5 |
| 14 | SSCUP | SBS7b | 0.55 (435) | 785 | DBS1 | 1.0 (76) | 76 |
| 15 | SSCUP | SBS6 | 0.32 (214) | 672 | - | - | ≤ 5 |
| 16 | SSCUP | SBS1 | 0.43 (342) | 797 | - | - | ≤ 5 |
| 17 | SSCUP | SBS87 | 0.25 (36) | 144 | - | - | ≤ 5 |
| 18 | SSCUP | SBS1 | 0.43 (571) | 1332 | - | - | ≤ 5 |
| 19 | SSCUP | SBS1 | 0.40 (175) | 437 | - | - | ≤ 5 |
| 20 | SSCUP | SBS1 | 0.29 (557) | 1891 | - | - | ≤ 5 |
| 21 | SSCUP | SBS1 | 0.51 (118) | 232 | - | - | ≤ 5 |
| 22 | SSCUP | SBS1 | 0.50 (390) | 787 | - | - | ≤ 5 |
| Control 1 | Cutaneous SCC | SBS7b | 0.26 (2140) | 8253 | DBS1 | 0.86 (229) | 265 |
| Control 2 | Cutaneous SCC | SBS7b | 0.41 (1454) | 3526 | DBS1 | 0.87 (120) | 138 |
| Control 3 | Cutaneous SCC | SBS7b | 0.26 (1757) | 6875 | DBS1 | 0.95 (190) | 199 |

**Supplemental Table 1** – Mutational signature analysis for SCCUP cases and cutaneous SCC controls with primary SBS and DBS mutational signature attribution. Proposed etiologies for identified primary SBS mutational signatures: SBS1 – Spontaneous deamination of 5-methylcytosine (clock-like signature); SBS7b – Ultraviolet light exposure; SBS6 - Defective DNA mismatch repair; SBS87 - Thiopurine chemotherapy treatment. Proposed etiologies for identified primary SBS mutational signatures: DBS1 – (Ultraviolet light exposure).
